# Innovative Therapeutic Approaches for Huntington’s Disease: From Nucleic Acids to GPCR-Targeting Small Molecules

**DOI:** 10.3389/fncel.2021.785703

**Published:** 2021-11-26

**Authors:** Hidetoshi Komatsu

**Affiliations:** ^1^Business Strategy, Kyowa Pharmaceutical Industry Co., Ltd., Osaka, Japan; ^2^Department of Biological Science, Graduate School of Science, Nagoya University, Nagoya, Japan

**Keywords:** antisense oligonucleotides, disease-modifying, G protein-coupled receptor, GPR52, Huntington’s disease, huntingtin lowering therapy, RNA interference

## Abstract

Huntington’s disease (HD) is a fatal neurodegenerative disorder due to an extraordinarily expanded CAG repeat in the huntingtin gene that confers a gain-of-toxic function in the mutant protein. There is currently no effective cure that attenuates progression and severity of the disease. Since HD is an inherited monogenic disorder, lowering the mutant huntingtin (mHTT) represents a promising therapeutic strategy. Huntingtin lowering strategies mostly focus on nucleic acid approaches, such as small interfering RNAs (siRNAs) and antisense oligonucleotides (ASOs). While these approaches seem to be effective, the drug delivery to the brain poses a great challenge and requires direct injection into the central nervous system (CNS) that results in substantial burden for patients. This review discusses the topics on Huntingtin lowering strategies with clinical trials in patients already underway and introduce an innovative approach that has the potential to deter the disease progression through the inhibition of GPR52, a striatal-enriched class A orphan G protein-coupled receptor (GPCR) that represents a promising therapeutic target for psychiatric disorders. Chemically simple, potent, and selective GPR52 antagonists have been discovered through high-throughput screening and subsequent structure-activity relationship studies. These small molecule antagonists not only diminish both soluble and aggregated mHTT in the striatum, but also ameliorate HD-like defects in HD mice. This therapeutic approach offers great promise as a novel strategy for HD therapy, while nucleic acid delivery still faces considerable challenges.

## Introduction

Huntington’s disease (HD) is an autosomal dominant neurodegenerative disease, typically showing abnormal movement (predominantly chorea), cognitive impairment, and psychiatric features. The average age at onset is 40 years, followed by an inexorable clinical deterioration and average survival of 15 to 20 years (Ghosh and Tabrizi, 2018). Juvenile HD begins before the age of 21 and then worsens rapidly, usually eventuating in death 10 years or so from the motor onset (Quigley, 2017). HD prevalence fluctuates with up to a 10fold difference across the world (Rawlins et al., 2016). The prevalence studies show a worldwide prevalence of 2.7 per 100,000 with the highest rates in Western countries (Pringsheim et al., 2012; Rawlins et al., 2016). Current remedies are limited to symptomatic treatments, such as quetiapine in the treatment of behavioral symptoms of HD (Alpay and Koroshetz, 2006), as no treatment has been demonstrated to forestall or decelerate the disease progression.

Huntington’s disease pathology is characterized by abnormal protein aggregation and its spread in brain, as seen in Alzheimer’s disease, Parkinson’s disease, and amyotrophic lateral sclerosis (ALS; Boland et al., 2018). This is attributed to an expanded cytosine-adenine-guanine (CAG) repeat in exon 1 of the huntingtin gene (HTT) on chromosome 4. This genetic change encodes an abnormally long polyglutamine (polyQ) sequence near the amino terminus of the huntingtin protein (HTT). This protein has toxic features with dysfunction and death of neurons. Striatal GABAergic medium spiny neurons (MSNs) are particularly vulnerable to mutant huntingtin-induced toxicity, while HD has been recognized as a disorder of the entire body and brain (Bates et al., 2015). Its genetic pathogenesis allows for diagnostic and predictive testing for the disease. Furthermore, the genetic certainty of HD enables us to provide appealing genetic models to study disease mechanisms and therapeutic approaches.

Intensive research has provided substantial insights into the pathological mechanisms in HD. Given the monogenic nature of HD in which the pathology is attributed to production of mutant huntingtin protein (mHTT), lowering of mHTT has emerged as a promising therapeutic approach. Clinical trials are currently planned or underway for novel therapeutics for HD, mostly gene silencing or mHTT-lowering medicines aimed at reducing production of the mHTT. However, delivery of these HTT lowering therapies, such as small interference RNAs and antisense oligonucleotides (ASOs), has proven to be a considerable challenge. It needs injection directly into the central nervous system (CNS) that places a great burden on patients. In addition, these agents are difficult to reach the human brain, especially the striatum in which the most striking neuronal loss is observed. Naked siRNAs do not penetrate the blood-brain barrier (BBB) as well as the cell membrane, and therefore need viral vectors to transport siRNA into neurons through bilateral direct injection into the striatum (Chen et al., 2020; Wang et al., 2021). Another critical issue is that human brain is distinctive for its anatomical complexity. Thus, promising findings from animal models do not necessarily lead to successful outcomes in human. Since human brain is approximately 3,000 times larger than mouse brain, any oligonucleotide delivery into the brain would exhibit a totally different pattern of distribution throughout the parenchyma (Pouladi et al., 2013).

G protein-coupled receptors (GPCRs), also known as seven-transmembrane domain receptors, are one of the most widely exploited targets for therapeutics and account for nearly 30% of the Food and Drug Administration (FDA)-approved drug targets. In particular, most neuropharmacological drugs are well known to control GPCR activity in the CNS (Hauser et al., 2017). Among them, GPR52 is an orphan GPCR exclusively expressed in the brain, especially in the striatum (Komatsu et al., 2014), and represents a potential therapeutic target for HD (Komatsu, 2021; Wang et al., 2021). Deletion or knockdown of GPR52 alleviates HD-like phenotypes in the animal models and patient induced pluripotent stem cell (iPSC)-derived neurons (Yao et al., 2015; Song et al., 2018). More recently, highly potent, specific, and BBB-penetrating GPR52 antagonists have been discovered through high-throughput screening and subsequent structure-activity relationship (SAR) study. Furthermore, these antagonists not only reduce mHTT levels but also ameliorate HD-associated phenotypes in HD mice (Wang et al., 2021). This review will cover the mechanisms of HD pathogenesis, current clinical trials, and innovative approaches that aim to prevent or slow the disease progression.

## Pathological Mechanisms in Huntington’s Disease

Huntingtin gene encodes a large protein that is broadly expressed throughout the adult body with the highest level in the brain and has multiple interaction domains (Sharp et al., 1995; Sayer et al., 2005), playing a role as a scaffolding protein that clusters many binding partners (Cattaneo et al., 2005; Zuccato et al., 2010). HTT is localized in the cytoplasm, endosomes (Pal et al., 2006; Atwal et al., 2007), and the nuclear matrix (Atwal et al., 2007). Although wild-type HTT engages distinct signaling events, such as axonal transport, vesicular trafficking, cell division, and transcriptional regulation, and is indispensable for early embryonic development (Bates et al., 2015; Saudou and Humbert, 2016), its normal function remains largely elusive. Deletion of mouse HTT prior to neural development results in embryonic lethal. Conditional deletion of HTT in the forebrain in the early postnatal period leads to a progressive degenerative neuronal phenotype (Dragatsis et al., 2000). However, the depletion fails to cause adult neurodegeneration or animal death at more than 4 months of age, whereas the knockout mice die at 2 months of age of acute pancreatitis owing to the degeneration of pancreatic acinar cells (Wang et al., 2016). These findings imply that wild-type HTT reduction is unlikely to be harmful in adult patients treated with HTT lowering therapies.

Expansion of the CAG repeats of the HTT gene to 40 or more causes the adult onset with a mean age of 40 years, whereas patients with juvenile HD, carrying a mutation with more than 55 CAG repeats, show much more aggressively progression (Bates et al., 2015). The CAG repeat expansion and subsequent outcomes have been confirmed in cultured fibroblasts from HTT mutation carriers (Seong et al., 2005; Reis et al., 2011; HD iPSC Consortium, 2012), up to 15 years before the onset of HD symptoms (Paulsen et al., 2008). The CAG repeat length in HTT determines the degree of severity of the disease and is also a crucial determinant of the rate of HD pathogenesis that leads to the distinctive features of motor dysfunctions. The CAG size accounts for more than 56% of the variation found in the age at motor onset (Gusella et al., 2014). Most of the remaining variation can be attributed to genetic differences among the patients that affect the rate of HD pathogenesis. For instance, TP53, MAP2K6, MAP3K5, GRIN2A, GRIN2B, NPY, NPY2R, and ADORA2A, have been considered as potential genetic modifiers of HD (Gusella et al., 2014). The genome-wide unbiased analyses may provide new clues to disease pathways or processes as therapeutic targets competent for slowing the progression.

As already mentioned, mHTT has an aberrant elongated polyQ tract near the amino terminus. This leads to the accumulation of protein aggregates that initiates a cascade of pathogenic events. The biological and pathological features include transcriptional and synaptic dysfunctions, aberrant axonal trafficking and proteostasis, imperfect nuclear pore complex, oxidative stress, and mitochondrial malfunction (Bates et al., 2015; Grima et al., 2017). The full-length mHTT expression in animal models is well known to elicit HD-like neuropathology and behaviors, including striatal atrophy and motor deficits (Slow et al., 2003). The proteolytic cleavage of the mHTT by endoproteases, including caspases and calpains, is also found to produce N-terminal mHTT fragments that can generate neurotoxicity (Ratovitski et al., 2009; Landles et al., 2010). Indeed, the mHTT fragments have been isolated from HD postmortem brains (Lunkes et al., 2002). Furthermore, the polyQ-containing domain of the HTT protein or exon 1 HTT sufficiently provokes progressive neurological phenotypes in mice (Mangiarini et al., 1996). Intriguingly, CAG repeat length-dependent aberrant splicing of exon 1 HTT yields the pathogenic exon 1 protein (Sathasivam et al., 2013). On the other hand, the CAG-expanded HTT mRNA by itself may be responsible for HD toxicity (Rue et al., 2016). These findings suggest that targeting the leading cause of disease, namely, mutated HTT, could abrogate all these potential underlying pathophysiological mechanisms.

## Diagnosis

Huntington’s disease is generally diagnosed based on findings from clinical evaluation, patient history (including any family history) and, in most cases, genetic testing for the presence of the CAG expansion in HTT. The characteristic features are motor dysfunction (most typically chorea), cognitive impairment (such as deficits in attention and emotion recognition), and psychiatric symptoms (typically apathy and blunted affect). In some cases, especially if a patient’s family history and genetic testing are inconclusive, brain imaging, such as computed tomography (CT) or magnetic resonance imaging (MRI), can support the diagnosis. As the disease progresses, these scans typically show symmetrical striatal atrophy of the caudate and putamen, a concomitant increase in size of the fluid-filled lateral ventricle, and often, to a lesser degree, atrophy in cerebral cortical gray matter and subcortical white matter. These atrophy patterns do not necessarily indicate HD, because they can occur in other disorders. A person has normal findings on a CT or MRI scan, while showing early symptoms of HD (Bates et al., 2015).

The Unified Huntington’s Disease Rating Scale (UHDRS) is a standardized test used to quantify the severity of HD. This test measures the patient’s abilities in four general areas: motor control, cognitive symptoms, behavioral symptoms, and function in day-to-day life. UHDRS allows us to determine how much of an impact a drug or treatment is having on an individual’s HD (Siesling et al., 1998). The different areas of the rating scale can be administered separately. A UHDRS total motor score (TMS) is strongly supportive of the diagnosis and is formed of 15 items. The different items of the UHDRS-TMS include chorea, dystonia, parkinsonism, motor performance, oculomotor function, and balance (Mestre et al., 2018). Diagnosis of motor onset of manifest HD is currently made based on the UHDRS-TMS (Reilmann and Schubert, 2017). Furthermore, a more extensive series of diagnostic classifications have been proposed by considering results of cognitive function, natural history, and neuroimaging studies. The more formal definitions based on natural history that have been proposed are as follows: a premanifest or presymptomatic stage, followed by a prodromal phase and manifest HD (Reilmann et al., 2014). Initially, a period occurs in which individuals exhibit no clinical symptoms of HD and are therefore termed “presymptomatic,” typically up to 10 to 15 years before the onset. Individuals may then enter the “prodromal” period, during which subtle signs and symptoms are observed. Manifest HD shows slow progression of motor and cognitive impairments, accompanied by chorea often prominent early but plateauing or even diminishing later. Fine motor impairments, such as incoordination, bradykinesia, and rigidity, progress more steadily (Reilmann et al., 2014).

## Innovative Therapeutic Approaches

### RNA-Based Approaches

The current innovative therapeutic approaches offer great promise as HD modifiers. Most of these approaches focus on targeting mHTT production pathways to tackle the most proximal cause of HD pathogenesis. They include RNA-targeted therapeutics, such as ASOs and RNA interference (RNAi), that inhibits the mHTT expression at the post-transcriptional level. These approaches trigger cleavage, degradation, or transcriptional repression of mutant HTT expression.

#### Antisense Oligonucleotide

Antisense oligonucleotides are short single stranded oligomers constituted of chemically modified nucleotides specifically designed as complementary to target mRNA (Rinaldi and Wood, 2018). The hybridization of the mRNA target with the ASO gets signaled for degradation by endogenous enzymes, such as RNase H1, or blocks the target RNA’s functions, leading to translation arrest or modulation of RNA processing. ASOs are incapable of crossing the BBB and therefore require direct CNS administration (Bennett et al., 2017). Recent drug approvals include nusinersen, an ASO for the treatment of spinal muscular atrophy (Messina and Sframeli, 2020), demonstrating the potential of ASOs for the treatment of a neurological disease.

Antisense oligonucleotides have been demonstrated to exhibit long-lasting reduction of both HTT mRNA and its protein throughout the CNS in multiple transgenic animal models of HD (Keiser et al., 2016). These effects are dose-dependent with a maximal reduction of more than 75%, where the modest reduction of more than 35% correlates with phenotypic and survival benefits (Kordasiewicz et al., 2012; Stanek et al., 2013; Southwell et al., 2018). HTTrx, also known as ISIS 443139 or RG6042, is an allele non-specific ASO (thereby targeting mHTT and wild-type HTT) developed by Ionis Pharmaceuticals. The design of the first clinical trial was a randomized, double-blind, multiple-ascending-dose, phase 1-2a study performed in patients with early HD (Tabrizi et al., 2019). The study patients were assigned to HTTrx or placebo within each of five dosing cohorts (10, 30, 60, 90, or 120 mg). The dose selection was made according to preclinical experiments in rodents and non-human primates that related the dose levels to decline in mHTT concentration. Each patient received four bolus intrathecal injections of HTTrx or placebo (artificial cerebrospinal fluid) at 4-week intervals followed by a 4-month untreated follow-up period. The main objectives were evaluation of the safety and HTTrx pharmacokinetics in cerebrospinal fluid (CSF). Other objectives include the effect of HTTrx on mHTT concentration of in CSF and functional assessments. Overall, HTTrx was well tolerated, showing no serious adverse events. HTTrx administration resulted in significant dose-dependent decline in CSF mHTT. The two highest doses, 90 and 120 mg, showed a mean decline of 40% in CSF mHTT, in which the steady-state maximal decline was not reached during this trial. The preclinical pharmacokinetics/pharmacodynamics model suggests that 40% decline in CSF mHTT is equivalent to 55–70% drop in cortical mHTT and 20–35% drop in striatal mHTT, and 60% decline in CSF mHTT reflect 70–85% drop in cortical mHTT and 35–50% drop in striatal mHTT (Smith and Tabrizi, 2020). In this trial, there are no meaningful differences in functional, cognitive, psychiatric, and neurological clinical outcomes between placebo and HTTrx treatment groups (Tabrizi et al., 2019). This investigation may be not designed or sufficiently powered to evaluate the effect of HTTrx on clinical outcomes in HD because the disease progresses slowly with changes occurring over years, not months.

Taking into consideration the potentially aversive effects linked with downregulated wild-type HTT levels, selective reduction of mHTT with preservation of wild-type HTT levels may be a more favorable approach than unselective knockdown of both HTT alleles. Approximately two-thirds of HD patients with European ancestry possess either single-nucleotide polymorphism (SNP) of the HTT gene, rs362307 (SNP1), rs362331 (SNP2), or both SNPs in phase with the CAG repeat expansion, highlighting these SNP variants as promising therapeutic targets for most of the HD patients (Lombardi et al., 2009; Kay et al., 2014; Svrzikapa et al., 2020). Wave Life Sciences has initiated phase 1b/2a trials with intrathecal allele-selective ASOs, WVE-120101 (PRECISION-HD1, ClinicalTrials.gov: NCT03225833) and WVE 120102 (PRECISION-HD2, ClinicalTrials.gov: NCT03225846), which specifically target the U variant of SNP1 and SNP2, respectively, in mutant HTT mRNA transcripts. In the PRECISION-HD2 trial, WVE 120102 reduced CSF mHTT by 12.4%, whereas total CSF HTT and neurofilament light (NfL) remained unaltered. No results have been disclosed on the PRECISION-HD1 trial (Rodrigues and Wild, 2020). Only a select group of HD patients who meet the SNP-specific inclusion criteria will be eligible for each trial.

#### RNA Interference

RNA interference-based approaches employ intrinsic and evolutionarily conserved mechanisms in the cytosol to promote targeted mRNA degradation. Potential modalities include siRNAs (DiFiglia et al., 2007), artificial microRNA (miRNA; McBride et al., 2008), and short-hairpin RNA (shRNA; Harper et al., 2005). Double-stranded RNA shows slow diffusion and inefficient brain uptake, thereby requiring viral vectors, including Adeno-associated virus (AAV), to express the RNAi, as seen in a gene therapy approach (Franich et al., 2008; Miniarikova et al., 2016). The RNAi binds mature mRNA from the HTT gene, demonstrating promising results in HD models (Boudreau et al., 2009; McBride et al., 2011).

The biotechnology company, uniQure, has begun a phase 1/2 trial to investigate the safety, tolerability, and efficacy of a one-time treatment of AMT-130 in HD patients (ClinicalTrials.gov: NCT04120493) (Rodrigues and Wild, 2020). AMT-130 consists of an AAV5 vector with an artificial microRNA (AAV5-miHTT) specifically tailored to silence both the mutant and wild-type HTT genes in an indiscriminate manner. This clinical trial is a multi-center, randomized, sham-controlled, double-blind study. It will consist of 3 treatment arms: the low and high dose patients receiving a single total dose of 6 × 1012 and 6 × 1013 genome copies, respectively, of AAV5-miHTT by an MRI-guided convection-enhanced delivery; and the imitation surgery arm in which participants will receive bilateral partial thickness burr holes with no intrastriatal injections. This trial will take about 5 years, where participants will be blinded to treatment assignment for 18 months, followed by a 3.5-year period for an unblinded treatment. The primary end point will be safety at 18 months, and the secondary end point will be CSF biomarkers and expression levels of the vector DNA and microRNA at 60 months. Other outcomes include biofluid and imaging biomarkers, together with clinical scales, such as the UHDRS motor, cognitive, behavior and functional subscales, HDQLIFE and Hospital Anxiety and Depression Scale (HADS), the Neuro-QoL, quantitate motor assessments (Q-Motor), and the Huntington’s Disease Cognitive Assessment Battery (HD-CAB; Rodrigues and Wild, 2020). This study is the first clinical trial using an AAV-mediated gene therapy in HD and will allow us to learn more about the feasibility, safety and effectiveness of this approach.

#### Issues and Challenges

##### Delivery of Antisense Oligonucleotides

Antisense oligonucleotides do not penetrate the BBB but can be solubilized in CSF and, therefore, can be delivered directly into the CNS *via* an intrathecal administration. Following administration, they are distributed throughout the brain parenchyma and then incorporated into neurons and glia. In contrast to siRNAs, ASOs do not necessitate a viral vector and cell transformation. Therefore, the repeated administration is needed to maintain therapeutic levels, with adverse events related to injection into the CSF. ASOs follow pharmacokinetics in a dose-dependent manner, allowing for regulated titration of HTT reduction.

The distribution of ASOs is affected by various elements, including CSF dynamics and clearance paths, volume of the injected bolus, and anatomy of the intrathecal space (Wolf et al., 2016). Indeed, the highest levels are observed in sites adjacent to the CSF, indicating that passive diffusion is strongly associated with drug distribution. Active transport of ASOs also may be involved in the distribution because neuronal populations containing higher levels of ASO even in areas with overall low ASO levels are found (Keiser et al., 2016). In addition, ASOs are difficult to reach the striatum that is the most vulnerable brain region in HD. Administration of lumber intrathecal bolus doses of HTTrx in non-human primates exhibited a 50% decline in cortical HTT but a 15–20% decline in striatal HTT (Leavitt et al., 2016), suggesting that the striatal HTT reduction in humans is much lower than in the cortex due to the CSF dynamics.

##### Delivery of RNA Interference

Viral vectors, such as recombinant AAVs and lentiviruses (LVs), are usually used to convey siRNAs to neurons, since naked siRNAs do not penetrate the cell membrane or the BBB. AAVs and LVs are non-pathogenic and non-replicating and elicit a minimal immune response. AAVs stay as nuclear episomes without integrating into the host genome, leading to stable gene expression at higher levels than LVs (Chen et al., 2018). Therefore, a single administration of siRNAs could theoretically exhibit a permanent action. This is advantageous but at present irreversible without antidotes.

RNA interference expression can be normally achieved in animal models by direct injection into the target brain. However, to achieve this in humans will necessitate stereotaxic neurosurgery, which is highly invasive and at high risk for death and infection. Therefore, RNAi that can be delivered to the CNS through peripheral administration represents a promising alternative. Some AAV serotypes, such as AAV7, AAV8, and AAV9, have been shown to have the ability to transduce neurons as well as other cells in the brain. Among them, AAV9 displays a remarkable ability to transduce not only neurons but also parenchymal brain cells (Manfredsson et al., 2009; Aschauer et al., 2013; Hammond et al., 2017). Furthermore, Deverman et al. (2016) produces AAV-PHP.B, an AAV9 capsid mutant, that crosses the BBB and transduces brain cells with approximately 40-fold greater efficiency than AAV9. In recent years, a lipid nanoparticle (LNP) has been demonstrated to be highly effective in transporting nucleic acid constructs to the CNS (Cullis and Hope, 2017). Hirunagi et al. (2021) have shown that LNP-mediated delivery of the CAG repeat-targeting siRNA specifically downregulates polyQ-expanded androgen receptor levels in the brain of spinal and bulbar muscular atrophy (SBMA) mice *in vivo*. These novel vectors and LNPs may offer a potential solution for non-invasive gene therapy, including CRISPR/Cas9-mediated genome editing, for the treatment of HD in the future.

### Small-Molecule Approaches: GPR52 Antagonist

#### Discovery of GPR52 as a Novel Therapeutic Target for Psychiatric Disorders

G protein-coupled receptors are one of the most intensively investigated drug targets, largely owing to substantial involvement in pathophysiology, pharmacological tractability, and accessibility for small molecule drug discovery (Hauser et al., 2017). GPCRs can be divided into two types of receptors, odorant/sensory and non-odorant. More than 80% of the non-odorant GPCRs are expressed in the CNS. Approximately 40% of this family of membrane proteins show rich and relatively specific expression in the CNS (Vassilatis et al., 2003; Regard et al., 2008; Komatsu et al., 2014). For instance, dopamine, serotonin, glutamate, and acetylcholine receptors, all of which are well-known neuropharmacological targets, are exclusively expressed in the brain. These metabolic receptors cause slow synaptic transmission necessary for exerting antipsychotic actions (Greengard, 2001). Therefore, there is no doubt that non-odorant GPCRs play a crucial role in CNS drug development today.

Dopamine plays an essential role in psychiatric disorders, such as schizophrenia and bipolar disorder, since all commonly prescribed antipsychotics exhibit antagonistic activity against Gi/o-coupled dopamine D2 receptors enriched in the striatum (Seeman, 2006). The striatum is strongly innervated by the ventral tegmental area (VTA), the origin of the dopaminergic mesolimbic pathway, and serves a central role in manifestation of psychiatric disorders (Rice et al., 2016; Carta et al., 2019). Comprehensive transcriptome analysis of non-odorant GPCRs revealed that GPR52 is abundantly expressed in human and rodent striatum and co-localizes with striatal dopamine D2 receptors (Komatsu et al., 2014). Furthermore, genetically engineered animal models, as well as biological and pharmacological studies have suggested that GPR52 has great potential of being a therapeutic psychiatric receptor (Komatsu et al., 2014; Setoh et al., 2014; Nishiyama et al., 2017a,b; Wang et al., 2020).

#### GPR52 Antagonists Reduce Mutant Huntingtin

An orally bioavailable small molecule that is distributed to the CNS would be the most attractive treatment for HD. Yao et al. (2015) first demonstrated the potential of blocking GPR52 for decreasing mHTT production levels as a disease modifier of HD. Initially, they identified GPR52 as a modulator of mHTT expressions in an siRNA screen *in vitro*. They also found that the lowering of GPR52 suppressed HD-associated phenotypes in both patient iPS-derived neurons and *in vivo* HD models of Drosophila (Yao et al., 2015). Song et al. (2018) demonstrated that heterozygous or homozygous embryonic elimination of GPR52 ameliorates disease-associated defects in HD mice. Direct injection of GPR52-targeting shRNAs into bilateral striata of adult HD mice markedly diminished not only GPR52 mRNA transcripts but also mHTT protein production in the striatum (Wang et al., 2021). These results might reflect that GPR52 activation might exacerbate HD progression.

**FIGURE 1:**
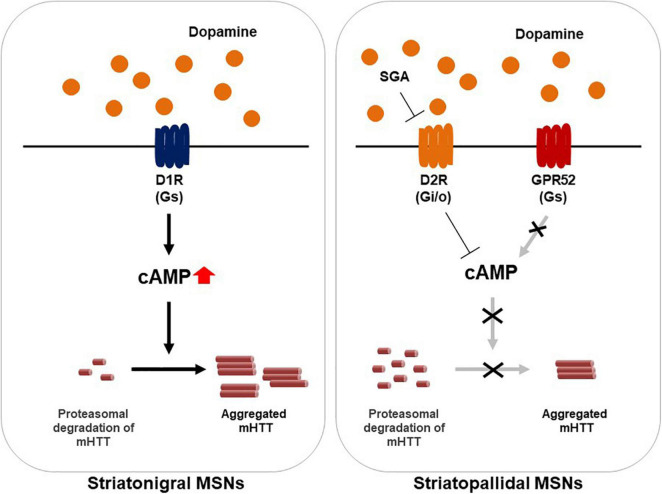
A model illustrating of GPR52 inhibition for the treatment of Huntington’s disease. In the striatum, GPR52 is expressed in the striatopallidal MSNs in which dopamine D2 receptor (D2R) is expressed. GPR52 inhibition or the antagonists leads to proteasomal degradation of the mutant huntingtin protein (mHTT). Oppositely, GPR52 activation *via* intracellular cAMP rise transports mHTT to the endoplasmic reticulum and protects against its degradation. Meanwhile, GPR52 is not expressed in the striatonigral MSNs where dopamine D1 receptor (D1R) is expressed. Second generation antipsychotics (SGA), such as quetiapine, blocks D2R-mediated signaling.

Several studies support the notion that GPR52 regulates HTT expression levels through a cAMP-dependent but PKA-independent signaling (Figure 1). In addition, the effect has been found to be mediated through elevated proteasomal degradation of mHTT by small GTPase Rab39B inactivation. Conversely, GPR52 activation results in mHTT translocation to the endoplasmic reticulum (ER) in which it can be protected from proteasomal degradation (Figure 1; Yao et al., 2015). While the role of cAMP pathway is complicated in HD and other GPR52-mediated cascades cannot be excluded, the fact that targeting GPR52 by genetic deletion or knockdown remarkably lessens mHTT levels and improves HD-related defects in multiple HD models is sufficiently compelling to justify GPR52 as a promising HD target.

More recently, Wang et al. (2021) synthesized a highly potent, specific, and BBB-penetrating GPR52 antagonist Comp-43 with an IC50 value of 0.63 μM through SAR study. Comp-43 markedly lowered both soluble and aggregated mHTT levels, as well as the striatal Iba1-positive microglia in HD mice *in vivo*. Furthermore, Comp-43 also improved the motor dysfunctions and disease-associated phenotypes in HD mice, including the reduction of neuroinflammation-related microgliosis and neuronal loss. Intriguingly, Comp-43 exerted no obvious influence on wild-type HTT production in wild-type mice (Wang et al., 2021). Taken together, these findings highlight the tremendous potential of pharmacological GPR52 inhibition for the treatment of HD.

## Conclusion

Tremendous advances have been made in understanding the clinical manifestations of HD with the identification of numerous potential therapeutic targets. While a number of clinical trials have been unsuccessful (Bashir, 2019), many more are in progress with the prospect of providing evidence for disease-modifying therapies. Among them, the current trials of mHTT-lowering hold great promise and may open up a new era for HD treatment at the proximal level. On the other hand, delivering ASOs, siRNAs, or genome editing reagents into the CNS is challenging and expensive. Therefore, small molecule drugs that lower mHTT levels are highly desired. GPR52 would be the best target for such small molecule drugs. Accumulating evidence suggests that transcellular spreading of mHTT may cause HD manifestations, as seen in other neurodegenerative disorders, such as Alzheimer’s and Parkinson’s diseases (Boland et al., 2018). If the propagation of mHTT begins in the striatum, decreasing the levels of mHTT exclusively in MSNs would be an effective approach to block its spread and slow the disease progression.

G protein-coupled receptors are located on the cell membrane and regulated by extracellular ligands, making them ideal targets for small molecule compounds. They control myriad physiological responses including neurotransmission, metabolism, hemostasis, reproduction, and immune function (Hauser et al., 2017). GPR52 is an orphan GPCR, though antipsychotic drug reserpine is identified as its surrogate ligand to elicit intracellular cAMP rise, indicating that this receptor is a Gs-coupled receptor. GPR52 is expressed in dopamine D2 receptor (D2R)-expressing MSNs of the basal ganglia, while largely expressed in dopamine D1 receptor (D1R)-expressing neurons in the medial prefrontal cortex (Figure 1). GPR52 represents a promising therapeutic target for the treatment of not only HD but also Parkinson’s disease (Russell et al., 2021), schizophrenia, and several other psychiatric disorders (Komatsu et al., 2014). For instance, GPR52 agonists are well-known to inhibit D2R signaling and activate D1R/NMDA receptors *via* intracellular cAMP accumulation, demonstrating antipsychotic-like and procognitive effects in rodents (Setoh et al., 2014; Komatsu, 2015; Nishiyama et al., 2017a; Wang et al., 2020).

A series of the druggable GPR52 antagonists have been discovered through approximately ten thousand compounds and subsequent SAR study. Wang et al. (2021) initially discovered compound F11 [(E)-1,7-diphenylhept-4-en-3-one] with IC50 value of approximately 5 μM. F11 consist of an α, β-unsaturated carbonyl group, a linker and terminal aryl-substitutes. Based on this compound, they explored the SAR and produced a novel, specific, and highly potent antagonist, or Comp-43. The SAR study demonstrates that α, β-unsaturated carbonyl group is an essential pharmacophore and the aryl substitutions on the two terminals form a π-π stacking interaction with the residues of GPR52 in the narrow binding cavity, which is consistent with recent findings on the high-resolution structure of human GPR52 (Lin et al., 2020). A computational study by molecular docking with GPR52 crystal structure have demonstrated that GPR52 antagonists enter the same binding site of GPR52 as the agonists, which is consistent with the SAR study (Wang et al., 2021). Thus, structure-based SAR investigation will facilitate the design of selective and potent GPR52 antagonists.

The endogenous ligand for GPR52 remains unknown and may not actually exist. The crystal structure of GPR52 uncovered a non-canonical mechanism of its extracellular loop (ECL2) acting as an internal agonist and accounting for constitutive activity of the receptor (Lin et al., 2020). Intriguingly, GPR52 negative allosteric modulator (NAM) and inverse agonists, including cannabinoid ligands Cannabidiol and O-1918, have been identified so far (Stott et al., 2021). These findings may help design various types of GPR52 modulators.

This review highlights GPR52 as a novel candidate target for HD therapy. However, some questions remain. First, it is unclear if GPR52 must be persistently inhibited. GPR52 knockout mice exhibited no change in body weight, brain morphology, and the travel distance. Meanwhile, they exhibited higher frequency of startle but not prepulse inhibition behaviors when treated with the NMDA receptor antagonist MK-801 (Komatsu, 2015), and enhanced the locomotor-stimulating effect of the ADORA2A antagonist istradefylline (Nishiyama et al., 2017a,b). GPR52 knockout mice also showed the increased time staying in the central region in the open-field test (Komatsu, 2015), as well as the increased novelty-induced locomotor activity (Nishiyama et al., 2017a,b). Taken all together, these findings indicate that GPR52 plays a key role in glutamatergic and dopaminergic synaptic transmissions and persistent GPR52 inhibition may elicit psychiatric manifestations. Second, GPR52-mediated action is non-allele-specific for HTT, leading to the downregulation of both the mutant and wild-type proteins (Wang et al., 2021). HTT serves as an essential protein involved in numerous biological processes. Non-allele-specific lowering of HTT would affect many of these processes. Third, it remains elusive whether inhibition of GPR52 signaling is enough to attenuate the progressive deterioration in HD. GPR52 is localized in the striatopallidal MSNs but not in all MSNs (Komatsu, 2015), which might result in a limited clinical impact (Figure 1).

In the last decade, we have seen a transition from symptomatic therapy to disease-modifying. Hence, the development of clinical and imaging biomarkers will be essential to accelerate the translation of HTT lowering therapies. Many HD pharmacodynamic biomarker candidates have been identified, including electrophysiologic clinical measures, biofluid biomarkers, and functional brain imaging (Weir et al., 2011). At present, only CSF biomarkers are the most useful for tracking disease progression in HTT lowering clinical trials (Byrne et al., 2018). Although neuroimaging techniques, particularly mHTT-specific positron emission tomography (PET) ligands could provide useful biomarkers for direct measurement of mHTT in brain, such ligands have not yet been established. Measurement of disease-associated biomarkers is a crucial element for clinical trial design. A biomarker indicative of neuronal damage or other pathological processes, such as neuroinflammation, will be instrumental for diagnosis, judging therapeutic efficacy, and tracking disease progression. Combination of biomarkers with predictive genetic HD testing can offer an opportunity to start treatment before symptom onset and deter neurodegeneration. We will see further expansion of HTT lowering therapies in the coming years, and much research on HD will refine and expand its goals in search of a treatment.

## Author Contributions

The author confirms being the sole contributor of this work and has approved it for publication.

## Conflict of Interest

HK is employed by Kyowa Pharmaceutical Industry Co., Ltd.

## Publisher’s Note

All claims expressed in this article are solely those of the authors and do not necessarily represent those of their affiliated organizations, or those of the publisher, the editors and the reviewers. Any product that may be evaluated in this article, or claim that may be made by its manufacturer, is not guaranteed or endorsed by the publisher.
